# Healthcare utilization and expenditures in patients with tricuspid regurgitation: A population-based cohort study

**DOI:** 10.1016/j.ijcha.2024.101495

**Published:** 2024-08-22

**Authors:** Ching-Hu Chung

**Affiliations:** Department of Medicine, Mackay Medical College, New Taipei City, Taiwan

**Keywords:** Tricuspid regurgitation, Heart failure, Healthcare costs, Health economics

## Abstract

**Background:**

Tricuspid regurgitation (TR) is the most common tricuspid valve (TV) condition. However, little is known about the prevalence, clinical significance, or economic impact of TR, including TR with comorbid heart failure (HF).

**Materials and Methods:**

Taiwan’s National Health Insurance Research Database was used to perform a retrospective cohort study about patients with TR. The study included patients over the age of 18 with TR who provided data from January 2017 to December 2019. The cohorts were divided into six groups based on whether significant TR was present (sTR) or not (nsTR), and whether HF was present (HF) or not present (noHF), or inconclusive (incHF).

**Results:**

This study included 21,051 patients with TR. Patients with nsTR-noHF had an annualized healthcare burden of 0.36 all-cause hospitalizations, 3.26 days length of stay (LOS), and NTD 66,834 in expenses. sTR led to significant increases in healthcare utilization and expenditures. The annualized economic burden for sTR-noHF patients increased to 1.03 all-cause hospitalizations, 10.75 days LOS, and NTD 210,842 in expenses. Patients with sTR and HF had significantly higher healthcare utilization and expenditures; patients with sTR-HF had an annualized economic burden of 2.46 all-cause hospitalizations, 33.18 days LOS, and NTD 480,711 in spending.

**Conclusion:**

TR patients with HF or sTR are more likely to be hospitalized, use more healthcare resources, and face higher financial burdens.

## Highlights

• Most cases of tricuspid regurgitation are not associated with heart failure.
• Tricuspid regurgitation patients associated with heart failure were older, sicker, and sought medical attention more frequently than those with no heart failure and inconclusive heart failure.
• Clinical significant tricuspid regurgitation patients are more likely to be hospitalized, use more healthcare resources, and face higher financial burdens.

## 1 Introduction

Tricuspid regurgitation (TR) is one of the most common valvular heart diseases, with over 80 % of cases diagnosed during a routine echocardiogram [1]. A study conducted in 2006 in the United States estimated that 1.6 million people suffer from moderate to severe TR [2]. The pathophysiology of TR involves the failure of the TV leaflets to coapt properly, which leads to backflow of blood. This backflow increases the volume load on the right atrium and right ventricle,which eventually leads to right heart failure if untreated. The primary etiological factors include conditions like rheumatic heart disease, infective endocarditis, and carcinoid syndrome, while secondary TR is often due to left-sided heart diseases, pulmonary hypertension, or atrial fibrillation [3], [4], [5].

Therapeutic strategies for TR depend on the severity and underlying cause of the regurgitation. Medical management has traditionally been the first-line treatment for primary TR, while secondary TR is treated based on the underlying cause [6]. Medical management typically includes the diuretics use manage symptoms of congestion and aldosterone antagonists to counter secondary hyperaldosteronism [7]. Treating the underlying condition is crucial in secondary TR. Surgical options like annuloplasty, valve repair, or valve replacement are considered when medical therapy is insufficient [8]. Annuloplasty, which involves suturing the TV annulus to a prosthetic ring, is often performed in cases of annular dilation [9]. Valve repair or replacement is indicated for primary valve abnormalities or when annuloplasty is not feasible [10].

This study assesses the incidence of TR in a nationwide sample of patients treated in Taiwan to provide context for the occurrence and management of TR, along with the healthcare utilization and expenditures of patients with varying severities of TR.

## 2 Materials and Methods

### 2.1 Data source and patient definition

The data for this retrospective, population-based study were obtained by accessing claim records from the National Health Insurance Research Database (NHIRD) between 2017 and 2019. The NHIRD contains information about all Taiwanese beneficiaries (23,603,121 individuals in 2019). Taiwan’s NHIRD is a public database accessible via a formal application approved by the Health and Welfare Data Science Center of the Ministry of Health and Welfare of Taiwan (https://dep.mohw.gov.tw/DOS/np-2500-113.html). The International Classification of Diseases (ICD) diagnosis and procedure codes (ICD-10) were used to determine patients with TR. Supplement Table 1 shows the detailed coding used in this study. The protocol of this study was authorized by the MacKay Memorial Hospital Institutional Review Board Taiwan R.O.C. (Protocol Number: 19MMHIS083e).

**Table 1:** Demographic data.

	noHF		HF		incHF	
	nsTR	sTR	nsTR	sTR	nsTR	sTR
**Total (N)**	19,672	504	661	155	48	11
**Age**						
11–20	186 (0.95 %)	7 (1.39 %)	0 (0.00 %)	0 (0.00 %)	0 (0.00 %)	0 (0.00 %)
21–30	820 (4.17 %)		16 (2.42 %)	0 (0.00 %)	0 (0.00 %)	0 (0.00 %)
31–40	1,330 (6.76 %)	14 (2.78 %)		4 (2.58 %)	4 (8.33 %)	0 (0.00 %)
41–50	2,380 (12.10 %)	45 (8.93 %)	25 (3.78 %)	13 (8.39 %)		0 (0.00 %)
51–60	4,347 (22.10 %)	88 (17.46 %)	41 (6.20 %)	12 (7.74 %)		0 (0.00 %)
61–70	5,292 (26.90 %)	108 (21.43 %)	98 (14.83 %)	30 (19.35 %)	9 (18.75 %)	7 (63.64 %)
71–80	3,345 (17.00 %)	119 (23.61 %)	172 (26.02 %)	50 (32.26 %)	17 (35.42 %)	
81–90	1710 (8.69 %)	95 (18.85 %)	222 (33.59 %)	36 (23.23 %)	14 (29.17 %)	4 (36.36 %)
≥91	262 (1.33 %)	28 (5.56 %)	87 (13.16 %)	10 (6.45 %)	4 (8.33 %)	0 (0.00 %)
Mean	60.57	68.45	76.85	72.72	76.06	76.55
Std	15.96	15.26	13.88	14.38	12.51	9.23
Median	62	69.5	80	75	77.5	79

**Elixhauser Comorbidity index**						
Mean	7.99	14.32	20.49	23.19	19.90	26.18
Std	6.59	8.91	7.03	8.84	6.48	6.94
Median	6	13	20	22	18.5	26

### 2.2 Cohort definitions

Patients with or without clinically significant TR who met the initial criteria above were divided into six mutually exclusive, collectively exhaustive groups based on whether significant TR was present (sTR) or not (nsTR), and whether HF was present (HF) or not present (noHF)(HF was identified as patients with ICD-10 coding I13.0, I13.2 and I50.#) [11], or inconclusive (incHF). The observation period of this study was the 12-month before the first TR diagnosis/invasive therapy and 6-month after first TR diagnosis/invasive therapy. HF diagnosis was defined as following: (1) not present HF (noHF): without HF diagnosis in the database during observation period, (2) present HF (HF): with at least one inpatient or two outpatient diagnoses of HF during observation period, and (3) inconclusive HF (incHF): with only one outpatient diagnosis of HF during observation period. Patients were considered to have clinically significant TR (sTR) if they had a history of pulmonary hypertension with ascites, edema, or hepatic insufficiency during the baseline or landmark periods, or if they had undergone TV surgery during that time.

### 2.3 Assessment

The Elixhauser Comorbidities Index (ECI) measures chronic disease severity, and the ECI score was calculated according to Walraven et al. [12]. We assessed healthcare utilization in patients with TR using emergency department visits, all-cause hospitalization, length of stay (LOS), congestive HF (CHF)–related hospitalizations (I09.9#, I11.0#, I13.0#, I13.2#, I25.5#, I42.0#, I42.5#, I42.6#, I42.7#, I42.8#, I42.9#, I43.#, I50.#, and P29.0#), and CHF–related LOS. We also investigated medical expenses to better understand the disease burden of TR patients.

### 2.4 Data analyses

SAS 9.1 (SAS Institute Inc., Cary, NC) was used to conduct data analyses. Variables were identified using the criteria described above. Frequencies or percentages were used to define categorical variables.

## 3 Results

### 3.1 Sample description

**Fig. 1:**
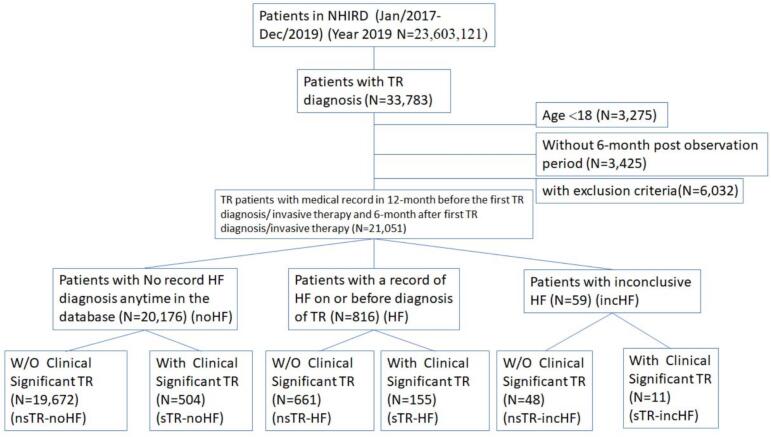
Attrition Diagram.

Patients who participated in the NHIRD between 2017 and 2019 comprised the study cohort. Our inclusion criteria identified 21,051 eligible TR cases between 2017 and 2019 (0.089 %) (Fig. 1). Of the 21,051 patients, 95.84 % (n = 20,176) were classified as noHF, 3.88 % (n = 816) as HF, and 0.28 % (n = 59) as incHF. These patients were further divided based on clinically significant TR criteria in six groups. In noHF group, 2.50 % (n = 504) was sTR-noHF and 97.5 % (n = 19,672) was nsTR-noHF. The sTR-HF was 19.00 % (n = 155) and nsTR-HF was 81.00 % (n = 661) among HF, while 18.64 % (n = 11) of sTR-incHF and 81.36 % (n = 48) of nsTS-incHF among incHF.

Patients in the HF and incHF cohorts were older than those in the noHF cohort, regardless of whether they had clinically significant TR or not. The average age of the nsTR-noHF cohort was 60.57 years, while the nsTR-HF and nsTR-incHF cohorts had average ages of 76.85 years and 76.06 years, respectively. The average age of the sTR-noHF cohort was 68.45 years, while that of the sTR-HF and sTR-incHF cohorts were 72.72 and 76.55 years, respectively. The nsTR-HF cohort had a higher ECI (20.49) than the nsTR-noHF (7.99) or nsTR-incHF cohorts (19.90) (Table 1). The HF cohort had a higher prevalence of almost all underlying diseases than those in the noHF and incHF cohorts (Table 2). The sTR-incHF cohort had a higher ECI score of 26.18 compared to the sTR-noHF (14.32) and sTR-HF cohorts (23.19) (Table 1). Patients with clinically significant TR scored significantly higher on ECI than those without. This was true for all TR patients, regardless of the HF diagnosis.

**Table 2:** The underlying diseases among these TR Patients.

	noHF		HF		incHF	
	nsTR	sTR	nsTR	sTR	nsTR	sTR
Congestive Heart Failure	3,863 (19.64 %)	208 (41.27 %)	655 (99.09 %)	152 (98.06 %)	47 (97.92 %)	11 (100.00 %)
Cardiac Arrhythmia	5,323 (27.06 %)	158 (31.35 %)	401 (60.67 %)	101 (65.16 %)	27 (56.25 %)	8 (72.73 %)
Valvular Disease	19,592 (99.59 %)	503 (99.80 %)	661 (100.00 %)	155 (100.00 %)	48 (100.00 %)	11 (100.00 %)
Pulmonary Circulation Disorders	2,96 (1.50 %)	85 (16.87 %)	101 (15.28 %)	23.23 %	8 (16.67 %)	0 (0.00 %)
Peripheral Vascular Disorders	450 (2.29 %)	35 (6.94 %)	50 (7.56 %)	9 (5.81 %)	4 (8.33 %)	≤3 (≤27.27 %)
Hypertension (Uncomplicated)	6,953 (35.34 %)	245 (48.61 %)	322 (48.71 %)	66 (42.58 %)	26 (54.17 %)	≤3 (≤27.27 %)
Hypertension (Complicated)	7,807 (39.69 %)	218 (43.25 %)	456 (68.99 %)	103 (66.45 %)	28 (58.33 %)	9 (81.82 %)
Paralysis	73 (0.37 %)	5 (0.99 %)	8 (1.21 %)	≤3 (≤1.94 %)	≤3 (≤6.25 %)	0 (0.00 %)
Other Neurological Disorders	14,871 (75.59 %)	445 (88.29 %)	580 (87.75 %)	138 (89.03 %)	42 (87.50 %)	11 (100.00 %)
Chronic Pulmonary Disease	3,310 (16.83 %)	142 (28.17 %)	274 (41.45 %)	59 (38.06 %)	22 (45.83 %)	5 (45.45 %)
Diabetes (Uncomplicated)	2,993 (15.21 %)	117 (23.21 %)	183 (27.69 %)	44 (28.39 %)	15 (31.25 %)	4 (36.36 %)
Diabetes (Complicated)	1,928 (9.80 %)	85 (16.87 %)	171 (25.87 %)	41 (26.45 %)	10 (20.83 %)	4 (36.36 %)
Hypothyroidism	395 (2.01 %)	17 (3.37 %)	17 (2.57 %)	10 (6.45 %)*	≤3 (≤6.25 %)	≤3 (≤27.27 %)
Renal Failure	1,269 (6.45 %)	88 (17.46 %)	237 (35.85 %)	67 (43.23 %)	15 (31.25 %)	7 (63.64 %)
Liver Disease	831 (4.22 %)	86 (17.06 %)	40 (6.05 %)	34 (21.94 %)***	4 (8.33 %)	≤3 (≤27.27 %)
Peptic Ulcer Disease (excluding bleeding)	2,716 (13.81 %)	110 (21.83 %)	112 (16.94 %)	43 (27.74 %)	14 (29.17 %)	4 (36.36 %)
AIDS/HIV	11 (0.06 %)	0 (0.00 %)	≤3 (≤0.45 %)	0 (0.00 %)	0 (0.00 %)	0 (0.00 %)
Lymphoma	32 (0.19 %)	≤3 (≤0.60 %)	4 (0.61 %)	≤3 (≤1.94 %)	≤3 (≤6.25 %)	0 (0.00 %)
Metastatic Cancer	149 (0.76 %)	13 (2.58 %)	15 (2.27 %)	≤3 (≤1.94 %)	0 (0.00 %)	0 (0.00 %)
Solid Tumor without Metastasis	1,139 (5.79 %)	57 (11.31 %)	53 (8.02 %)	16 (10.32 %)	≤3 (≤6.25 %)	0 (0.00 %)
Rheumatoid Arthritis Collagen	822 (4.18 %)	38 (7.54 %)	30 (4.54 %)	≤3 (≤1.94 %)	≤3 (≤6.25 %)	0 (0.00 %)
Coagulopathy	114 (0.58 %)	24 (4.76 %)	14 (2.12 %)	≤3 (≤1.94 %)	≤3 (≤6.25 %)	0 (0.00 %)
Obesity	0 (0.00 %)	0 (0.00 %)	0 (0.00 %)	0 (0.00 %)	0 (0.00 %)	0 (0.00 %)
Weight Loss	199 (1.01 %)	10 (1.98 %)	15 (2.27 %)	5 (3.23 %)	≤3 (≤6.25 %)	≤3 (≤27.27 %)
Fluid and Electrolyte Disorders	608 (3.09 %)	46 (9.13 %)	163 (24.66 %)	39 (25.16 %)	10 (20.83 %)	4 (36.36 %)
Blood Loss Anemia	104 (0.53 %)	9 (1.79 %)	28 (4.24 %)	7 (4.52 %)	≤3 (≤6.25 %)	≤3 (≤27.27 %)
Deficiency Anemia	531 (2.70 %)	15 (2.98 %)	25 (3.78 %)	10 (6.45 %)	≤3 (≤6.25 %)	≤3 (≤27.27 %)
Alcohol Abuse	60 (0.31 %)	17 (3.37 %)	≤3 (≤0.45 %)	7 (4.52 %)	0 (0.00 %)	≤3 (≤27.27 %)
Drug Abuse	31 (0.16 %)	≤3 (≤0.60 %)	≤3 (≤0.45 %)	≤3 (≤1.94 %)	0 (0.00 %)	0 (0.00 %)
Psychoses	160 (0.81 %)	12 (2.38 %)	8 (1.21 %)	≤3 (≤1.94 %)	0 (0.00 %)	0 (0.00 %)
Depression	1,295 (6.58 %)	46 (9.13 %)	44 (6.66 %)	10 (6.45 %)	5 (10.42 %)	0 (0.00 %)

### 3.2 Healthcare utilization outcomes for patients with TR

Healthcare utilization data for patients with TR were collected at the 12-month baseline and 6-month landmark periods (Table 3). CHF and all-cause emergency department visits, hospitalizations, LOS, and outpatient department (OPD) visits were all calculated. The sTR-HF group had the highest utilization pattern in CHF OPD visits. The sTR-incHF group had the highest utilization patterns in the inpatient department (IPD), LOS, and CHF/all-cause costs. The nsTR-incHF group higher annual healthcare costs (NTD 293,569) than those with nsTR-HF or nsTR-noHF (NTD 276,827 and NTD 66,834, respectively) in patient cohorts without clinically significant TR. In patient cohorts with clinically significant TR, patients with sTR-incHF had higher annual healthcare expenditures (NTD 769,909) than patients with sTR-HF or sTR-noHF (NTD 480,711 and NTD 210,842, respectively).

**Table 3:** Economic burden in TR Patients.

	noHF		HF		incHF	
	nsTR	sTR	nsTR	sTR	nsTR	sTR
CHF OPD case number (N)	3,493	166	625	142	44	9
CHF OPD visit (mean)	7.29	6.51	10.42	11.61	1.89	1.56
CHF OPD cost (mean)	11,584	19,794	24,846	92,332	6,707	11,252
Total OPD case number (N)	19,643	501	654	152	48	11
Total OPD visit (mean)	31.54	41.11	37.56	35.59	37.13	33
Total OPD cost (mean)	79,193	272,708	126,837	524,398	250,228	235,333
CHF IPD case number (N)	299	45	602	138	41	11
CHF IPD visit (mean)	1.43	1.18	1.87	2.17	1.88	2.73
CHF IPD LOS (mean, days)	15.30	14.91	17.33	25.42	16.10	58.73
CHF IPD cost (mean)	120,250	188,925	123,952	247,326	120,851	406,102
Total IPD case number (N)	3,831	237	639	149	45	11
Total IPD visit (mean)	1.85	2.19	2.69	2.92	2.51	4.27
Total IPD LOS (mean, days)	16.75	22.87	26.62	34.52	22.56	78.55
Total IPD cost (mean)	119,529	219,393	192,344	348,327	188,717	627,598
Annual Cost for all healthcare costs (mean)	66,834	210,843	276,828	480,711	293,569	769,909
Annual Cost for CHF (mean)	3,885	23,388	136,381	304,788	109,375	415,308
Annual Cost for TR (mean)	9,542	57,018	46410	156,495	311,76	223,734

### 3.3 Regression

Table 4 shows the regression results for each of the annualized interest outcome variables (total expenditures and LOS). The two main independent variables—HF and clinically significant TR—were statistically significant for expenditures and all-cause hospitalizations. A diagnosis of HF increased to NTD 122,119 (p <0.0001) in annualized expenditures and 5.60 days (p = 0.0043) in annualized LOS. Patients with clinically significant TR experienced an increase of NTD 485,014 (p <0.0001) in annualized expenditures and of 5.53 days (p = 0.4347) in annualized LOS. Age was statistically significant in expenditures and all-cause hospitalizations, but not in annualized LOS. Annualized expenditures for pulmonary hypertension, ascites, and edema were significantly higher (NTD 394,779, NTD 89,488, and NTD 18,000, respectively; p <0.0001). The ECI score varied significantly for all three outcome variables of interest.

**Table 4:** Regression results in TR Patients.

	Expenditures		All-cause hospitalizations		LOS	
	Estimate	p-value	Estimate	p-value	Estimate	p-value
Heart failure: 1 vs. 0	122,119	<0.0001	0.34	<0.0001	5.60	0.0043
TR severe: 1 vs. 0	485,014	<0.0001	−0.49	0.0372	5.53	0.4347
Age (per unit increase)	496	<0.0001	−0.01	0.0020	−0.06	0.1528
Gender (Male vs. female)	−6,656	<0.0001	−0.04	0.5182	−2.25	0.0931
Pulmonary hypertension: 1 vs. 0	394,779	<0.0001	−0.57	0.0405	−7.90	0.2319
Ascites: 1 vs. 0	89,488	<0.0001	0.36	0.1506	7.88	0.1777
Edema: 1 vs. 0	18,000	0.0258	0.16	0.3094	6.05	0.0967
Hepatic insufficiency: 1 vs. 0	15,220	0.5094	0.38	0.2080	8.95	0.2103
Elixhauser comorbidity index (per unit increase)	6,597	<0.0001	0.06	<0.0001	0.55	<0.0001

## 4 Discussion

This study used Taiwan’s NHIRD to analyze epidemiological and healthcare utilization data for TR and sTR patients from 2017 to 2019. TR cases comprised 0.089 % of the total population. In this study, patients with noHF outnumbered HF or incHF patients by a large margin. There were more patients with sTR-noHF than those with sTR-HF or sTR-incHF. Additionally, we looked at the healthcare utilization and annual expenditures of with or without clinically significant TR patients. Compared to the HF and noHF cohorts, the incHF cohort had higher healthcare utilization and annual expenditures; this was due to incHF patients’ high ECI scores and comorbidities. The noHF cohort had fewer comorbidities and lower healthcare utilization in comparison to the HF and incHF cohorts.

Despite the low prevalence of significant TR in the general population (∼0.5 % of patients who undergo echocardiographic testing), CHF and other valvular heart diseases are commonly associated with significant TR [13]. In another retrospective study, 23 % of patients with symptomatic HF had moderate to severe TR [14]. Among all patients with TR, the prevalence of clinically significant TR in our study (sTR-noHF=504, sTR-HF=155, and sTR-incHF=11) was 3.18 %, while the percentage of clinically significant TR among HF patients was 19.00 % (sTR-HF=155). These findings were consistent with those from previous studies [13], [14].

Several observational studies have found that patients with clinically significant TR and CHF have a longer LOS, more rehospitalizations, and higher mortality rates [13], [14], [15], [16]. Patients with clinically significant TR or HF had more IPD visits and higher medical costs (Table 3). Following the regression analysis, two major independent variables (HF and clinically significant TR) were statistically significant across expenditures and all-cause hospitalizations (Table 4). These findings reflect rising rehospitalization rates among patients with HF and clinically significant TR in Western countries and Asia. HF is associated with higher expenditures and all-cause hospitalizations along with longer LOS (Table 4). This indicates that HF is an important factor in the economic burden for patients with TR.

In the current study, the annual medical expenses associated with sTR-HF were 1.59 times greater than those associated with sTR-noHF (Table 3). This result was consistent with other claims database studies, which found a 1.41- or 1.15-fold increase in incremental annual medical expenditures in patients with HF and clinically significant TR [11], [17]. Compared to these two U.S. claims database studies, the annual LOS in sTR-HF were higher than those in sTR-noHF, and the incremental trend was similar; however, the sTR-HF cohort in the current study was associated with 34.52 days annual LOS, which was significantly higher than 4.31 days (Cork et al., 2020) and 7.1 days (Barker et al., 2021). The reason for this could be the difference in health insurance coverage between Taiwan and the United States. In Taiwan, health insurance covers almost all diseases; patients are only responsible for copayments, the upper limits of which are capped at 7 USD per OPD and 1,433 USD per IPD. Thus, Taiwan’s higher rates of outpatient and inpatient service utilization may be attributed to relatively low out-of-pocket expenses.

Although NHIRD data provided useful analyses, this investigation was limited by the database’s design. First, we were unable to assess disease severity due to a lack of information on self-payment for medications, laboratory data, and patients’ vital statistics (height, weight, etc.) is limited; therefore, we were unable to assess disease severity. Second, because the study design was solely based on the ICD-10-CM and ICD-10-PCs, coding errors, misclassifications, or differences between hospitals and physicians may have influenced our findings. Finally, some TR surgeries or procedures were not reimbursed in Taiwan from 2017 to 2019. The National Health Insurance Administration requires more than a year to compile and publish annual data.

## 5 Conclusions

In this population-based national study of patients with TR, we discovered that HF patients were older, sicker, and more likely to seek medical attention than noHF and incHF patients. Among these patients with clinically significant TR, patients with sTR-incHF were older, sicker, and sought medical attention medical treatment more frequently than patients with sTR-noHF and sTR-HF. Among TR types, nsTR-incHF / sTR-incHF had the highest healthcare costs. Future research should compare various treatment options and their clinical and economic outcomes in real-world settings.

## CRediT authorship contribution statement

**Ching-Hu Chung:** Writing – review & editing, Writing – original draft, Project administration, Methodology, Investigation, Funding acquisition, Formal analysis, Data curation, Conceptualization.

## Declaration of competing interest

The authors declare that they have no known competing financial interests or personal relationships that could have appeared to influence the work reported in this paper.

## Data Availability

The data underlying this study belong to the National Health Insurance Research Database (NHIRD) of Taiwan and cannot be made publicly available due to legal restrictions. However, the data are available through formal application to the Health and Welfare Data Science Centre at Ministry of Health and Welfare, Taiwan (https://dep.mohw.gov.tw/DOS/np-2500-113.html) and require a signed affirmation regarding data confidentiality. The authors have no special privilege of access to the database.
